# Complete Genome Sequence of Genotype VI Newcastle Disease Viruses Isolated from Pigeons in Pakistan

**DOI:** 10.1128/genomeA.00845-16

**Published:** 2016-08-18

**Authors:** Abdul Wajid, Shafqat Fatima Rehmani, Poonam Sharma, Iryna V. Goraichuk, Kiril M. Dimitrov, Claudio L. Afonso

**Affiliations:** a Institute of Biochemistry and Biotechnology (IBBt), University of Veterinary and Animal Sciences, Lahore, Pakistan; b Quality Operations Laboratory (QOL), University of Veterinary and Animal Sciences, Lahore, Pakistan; c Exotic and Emerging Avian Viral Disease Research Unit, Southeast Poultry Research Laboratory, U.S. National Poultry Research Laboratory, ARS, USDA, Athens, Georgia, USA

## Abstract

Two complete genome sequences of Newcastle disease virus (NDV) are described here. Virulent isolates pigeon/Pakistan/Lahore/21A/2015 and pigeon/Pakistan/Lahore/25A/2015 were obtained from racing pigeons sampled in the Pakistani province of Punjab during 2015. Phylogenetic analysis of the fusion protein genes and complete genomes classified the isolates as members of NDV class II, genotype VI.

## GENOME ANNOUNCEMENT

Avian paramyxoviruses (APMVs) are currently classified into 12 accepted serotypes (designated APMV-1 to -12) and one putative (APMV-13) serotype (1–6). APMV-1, also known as Newcastle disease virus (NDV), belongs to the genus *Avulavirus*, family *Paramyxoviridae*, order *Mononegavirales* (6). Virulent NDVs are devastating for domestic fowl and have vast economic and social importance (7). The virus has single-stranded negative-sense, nonsegmented RNA genome with lengths of 15,186, 15,192, and 15,198 nucleotides (nt) (7, 8). Although all Newcastle disease viruses represent a single serotype, wide genetic diversity has been identified among the isolates across the globe (9). An antigenic variant of the virus, referred to as pigeon paramyxovirus 1 (PPMV-1), is occasionally isolated in poultry but commonly isolated from pigeons (10). Limited genetic information exists for the pigeon viruses circulating on the Asian continent, with predominantly Chinese genome sequences available in the GenBank database.

Two virulent NDVs, designated pigeon/Pakistan/Lahore/21A/2015 and pigeon/Pakistan/Lahore/25A/2015, were isolated from racing pigeons sampled in Punjab province, Pakistan, in 2015. The birds showed clinical signs (torticollis, tremor, and paralysis) that were more prominent in one of them. The viruses were submitted to the Southeast Poultry Research Laboratory of the USDA in Athens, Georgia, USA, and further propagated in nine-day-old specific-pathogen-free embryonated chicken eggs. Next-generation sequencing was used to determine the complete genomes of the viruses. Viral RNA was isolated from the allantoic fluid using a QIAamp RNA viral mini kit (Qiagen, USA). NDV RNA was captured and enriched using three biotin-labeled oligonucleotide probes and Sera-Mag beads (GE Healthcare Life Sciences, USA). A Moloney murine leukemia virus reverse transcriptase kit (Thermo Scientific, USA) was used for reverse transcription. Illumina libraries were prepared from the cDNA products by employing the Nextera XT DNA library preparation kit (Illumina, USA). The concentration of the prepared libraries and their fragment size distribution were checked using Double-stranded DNA HS assay kit (Life Technologies, USA) on a Qubit Fluorometer 2.0 and Agilent high sensitivity DNA kit (Agilent Technologies, Germany) on a Bioanalyzer 2100 instrument, respectively. Paired-end sequencing (2 × 250 bp) of the generated libraries was performed on an Illumina MiSeq instrument using the 500-cycle MiSeq reagent kit version 2 (Illumina). Sequence data were assembled using MIRA version 3.4.0 (11) within a customized workflow on the Galaxy platform (12). Short gaps (122 nt and 17 nt) at the 3′ untranslated region of the nucleoprotein gene were closed using Sanger technology.

The viral genomes contained six genes in the order 3′-NP-P-M-F-HN-L-5′ with coding sequence lengths of 1,470 nt, 1,188 nt, 1,095 nt, 1,662 nt, 1,716 nt, and 6,615 nt, respectively. Phylogenetic analysis (data not shown) classified the viruses as members of class II, genotype VI. The viruses contained three basic amino acid residues between positions 113 and 116 of the fusion protein cleavage site and a phenylalanine at position 117 (_113_RQKR↓F_117_). Such a motif in the deduced amino acid sequence of the cleavage site is typical for virulent NDV isolates (13). The genomic sequences provided here represent viruses from a new subgenotype of genotype VI identified for the first time in Pakistan.

### Accession number(s).

The genome sequences of pigeon/Pakistan/Lahore/21A/2015 and pigeon/Pakistan/Lahore/25A/2015 have been deposited in GenBank under the accession numbers KX236100 and KX236101, respectively.
